# Prevalence and Temporal Trends of Female Genital Cutting Among Women in Sierra Leone

**DOI:** 10.1007/s00192-026-06644-1

**Published:** 2026-05-22

**Authors:** Marie Bangura, Christopher X. Hong

**Affiliations:** 1 Division of Gynecology, Beth Israel Lahey Hospital, 67 S. Bedford Street, Suite 300 West, Burlington, MA USA; 2 Department of Obstetrics and Gynecology, University of Michigan, Ann Arbor, MI USA

**Keywords:** Female genital cutting, Female genital mutilation, Prevalence, Sierra Leone

## Abstract

**Introduction and Hypothesis:**

Female genital cutting (FGC) remains prevalent in Sierra Leone, yet conventional cross-sectional analyses limit understanding of age-specific risk and long-term temporal trends. We aimed to estimate age-specific prevalence of FGC from 1984 to 2019 and to compare prevalence levels and trends before and after 2013 across selected age cohorts.

**Methods:**

We conducted a secondary analysis of cross-sectional data from the 2019 Sierra Leone Demographic and Health Survey, which included women aged 15–49 years. Participants reported FGC status and age at which FGC occurred. Using respondent age at survey and reported age at FGC, we reconstructed calendar-year–specific prevalence estimates for women aged 15, 20, 25, 30, and 35 years from 1984 to 2019. Weighted prevalence before (1984–2013) and after 2013 (2014–2019) was compared, and temporal trends were assessed using survey-weighted linear regression.

**Results:**

Among 14,644 respondents, the weighted prevalence of FGC was 87.3%. The median age at FGC was 12 years (interquartile range 9–15). Weighted prevalence was highest among women aged 35 years (94.2%) and lowest among those aged 15 years (72.2%), with a marked increase between ages 15 and 20. Significant declines in prevalence were observed across all age groups between the pre- and post-2013 periods, with the largest absolute reduction among 15-year-olds (−15.9%). However, no age group demonstrated a statistically significant change in the rate of decline after 2013.

**Conclusions:**

Prevalence of FGC in Sierra Leone has declined over time, particularly among younger cohorts, but remains high. Late adolescence and early adulthood represent a critical period of ongoing risk

## Introduction

Female genital cutting (FGC), also known as female genital mutilation and female circumcision, involves alteration or injury to the external female genitalia for nonmedical indications [1]. This practice affects an estimated 230 million girls and women worldwide, and is recognized by the World Health Organization (WHO) as a human rights violation [2]. As such, there are efforts to eradicate this practice [1, 3, 4]. Despite these efforts, prevalence of FGC remains high, especially in several African nations [5–7].

In Sierra Leone, the prevalence of FGC was assessed in 2013 and 2019 based on the national Demographic and Health Survey (DHS), a periodic, nationally representative household survey that collects high-quality data on the country’s population and health status. However, these surveys only provide two cross-sectional snapshots of FGC prevalence, limiting the ability to characterize year-by-year trends, particularly among younger age groups. In addition, DHS surveys sample women aged 15–49 from households rather than fixed birth cohorts, resulting in potential overlap of both individual respondents and age-defined population segments across successive surveys, which complicates temporal interpretation [8, 9]. More granular, temporally resolved data are therefore needed to better understand age-specific patterns of risk and to inform the timing and targeting of anti-FGC efforts.

In this study, we aim to estimate the prevalence of FGC among women aged 15, 20, 25, 30, and 35 in Sierra Leone from 1984 to 2019. Next, we compare the proportions and temporal trends of FGC before and after 2013 using a novel temporal analysis that incorporates self-reported FGC status and the reported age at which FGC occurred.

## Material and Methods

We performed a secondary analysis of cross-sectional data from the 2019 Sierra Leone Demographic and Health Survey (DHS). The population of Sierra Leone in 2019 was 7.73 million people [10]. The DHS included 13,399 households with 22,771 total respondents. The DHS is designed to provide up-to-date estimates of key demographic and health indicators to inform evidence-based programs and policies aimed at improving public health. The survey’s sampling procedures have been previously described [8]. Briefly, the DHS employed a two-stage stratified cluster sampling design based on the 2015 national census framework. In the first stage, 578 enumeration areas were selected using probability proportional to size. In the second stage, 24 households were randomly selected from each cluster, ensuring nationally representative coverage.

As part of the survey, all eligible women aged 15 to 49 years were administered a standardized Women’s Questionnaire, which collected data on fertility history, contraceptive knowledge and use, breastfeeding practices, and female genital cutting. Participants were asked whether they had undergone FGC and, if so, at what age the procedure occurred.

Unlike prior analyses that simply reported the proportion of respondents affirming FGC by broad age groups (e.g., 15–20 years), we aimed to generate more precise annual estimates of FGC incidence by incorporating both the respondent’s age at the time of the survey and the age at which FGC was reported to have occurred. For example, a respondent who was 20 years old in 2019 and reported undergoing FGC at age 10 was classified as having had the procedure in 2009. This method uses the cross-sectional data to retrospectively reconstruct the calendar year in which FGC was reported to have occurred. As such, the calendar-year-specific estimates are reconstructed prevalence patterns rather than prospectively observed annual prevalence.

Using these data for all respondents, we calculated the proportion of women aged 15, 20, 25, 30, and 35 who had undergone FGC in each calendar year. Sampling weights provided by the DHS were applied in all analyses to account for the two-stage stratified cluster sampling design and to ensure nationally representative estimates.

To examine trends over time, we compared the proportion of women who had undergone female genital cutting before and after 2013. The year 2013 was selected because it corresponds to the last time DHS was performed in Sierra Leone, providing a methodological benchmark for comparison. No major legislation regarding FGC was enacted during this period; therefore, this cut off does not evaluate the impact of any policy changes. Differences in weighted FGC prevalence between the 1984–2013 and 2014–2019 periods were assessed within each age group using survey-weighted linear regression models with cohort period as the primary exposure. Mean annual rates of change were estimated using linear regression of prevalence on calendar year, and differences in temporal trends before and after 2013 were evaluated using interaction terms between year and period. Statistical significance was assessed using Wald tests. A *p* value < 0.05 was considered statistically significant. All analyses were conducted using R statistical software (R Foundation for Statistical Computing, Vienna, Austria).

Ethical approval for this study was obtained from the Sierra Leone Ministry of Health and Sanitation and the DHS Program’s ethics review committee. All survey participants provided informed consent at the time of data collection. Authorization to use the data was requested from and approved by the Measure DHS Program. The de-identified dataset is publicly accessible through the DHS Program website.

## Results

Among the 15,574 women surveyed in the 2019 Sierra Leone Demographic and Health Survey, 14,644 (94.0%) responded to the question regarding FGC. Of these respondents, 13,046 (89.1%) reported having undergone FGC. After applying the DHS sampling weights to generate nationally representative estimates, the weighted prevalence of FGC was 87.3%.

**Fig. 1:**
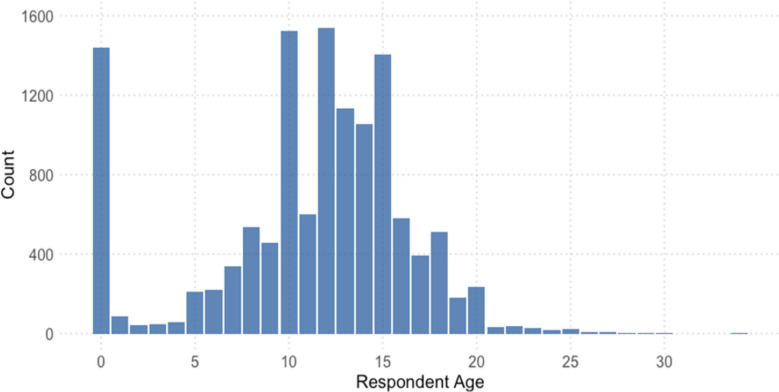
Respondent age at time of female genital cutting (FGC). Responses of FGC in infancy are coded as 0

**Fig. 2:**
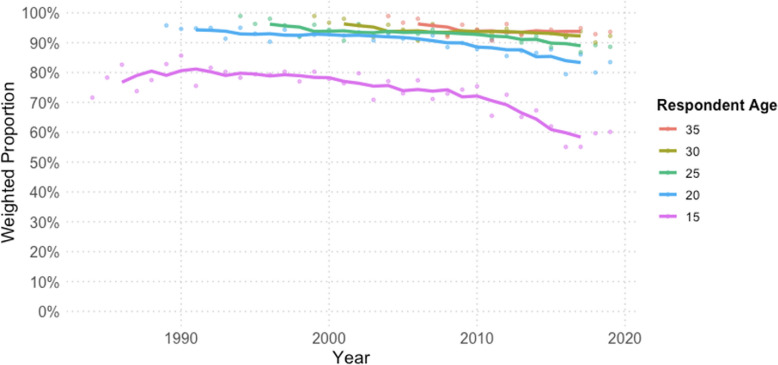
Prevalence of female genital cutting among respondents aged 15, 20, 25, 30, and 35 from 1984 to 2019. Lines represent 5-year moving averages from reconstructed estimates of prevalence

The median age at which FGC was performed was 12 years (interquartile range 9–15 years; range 0–34 years), although the distribution is bimodal and skewed to the right (Fig. 1). Notably, 9.2% of respondents reported undergoing FGC in infancy, or at 0 years. After applying sampling weights, the prevalence of FGC was highest among women aged 35 years (94.2%) and was progressively lower in successively younger age groups, reaching 72.2% among 15-year-olds (Fig. 2).

Across all respondent age groups, the weighted prevalence of FGC declined between the periods 1984–2013 and 2014–2019 (Table 1). The largest absolute decline was observed among respondents aged 15 years, with prevalence decreasing from 76.0% to 60.1% (−15.9%, *p* < 0.01). Smaller but statistically significant declines were also observed among older age groups: age 20 (−7.4%, *p* < 0.02), age 25 (−4.0%, *p* < 0.03), age 30 (−1.7%, *p* < 0.04), and age 35 (−1.1%, *p* < 0.05).

**Table 1:** Weighted prevalence and mean annual rate of change in weighted prevalence of female genital cutting among respondents age 15, 20, 25, 30, and 35

Respondent Age	Weighted Prevalence, Overall	Weighted Prevalence, 1984–2013	Weighted Prevalence, 2014–2019	Percent Difference, 1984–2013 vs. 2014–2019	*p*–value
35	94.2%	94.8%	93.7%	–1.1%	< 0.05
30	93.7%	94.3%	92.6%	–1.7%	< 0.04
25	92.2%	93.6%	89.6%	–4.0%	< 0.03
20	89.3%	91.3%	83.9%	–7.4%	< 0.02
15	72.2%	76.0%	60.1%	–15.9%	< 0.01
Respondent Age	Mean Annual Change, Overall	Mean Annual Change, 1984–2013	Mean Annual Change, 2014–2019	Percent Difference, 1984–2013 vs. 2014–2019	*p*–value
35	–0.2%	–0.5%	–0.1%	0.4%	0.30
30	–0.2%	–0.2%	–0.4%	–0.1%	0.80
25	–0.3%	–0.2%	–0.6%	–0.4%	0.47
20	–0.4%	–0.3%	–0.8%	–0.5%	0.19
15	–0.7%	–0.4%	–1.3%	–0.9%	0.35

Although all age groups demonstrated negative mean annual change in weighted FGC prevalence, differences in annual rates of decline between the two periods (1984–2013 vs 2014–2019) were not statistically significant for any age group (Table 1). The greatest apparent acceleration in decline was observed among 15-year-olds, whose mean annual decrease was −1.3% during 2014–2019 compared with −0.4% during 1984–2013; however, this difference did not reach statistical significance (*p* = 0.35).

## Discussion

In this secondary analysis of cross-section data of women aged 15 to 49 in Sierra Leone in 2019, the weighted prevalence of FGC was 87.3%. From 1984 to 2019, we observed marked differences in the weighted prevalence of FGC by age group, with the highest weighted prevalence observed among respondents aged 35 years (94.2%) and a progressively lower prevalence in younger age groups. Notably, prevalence increased substantially from 72.2% at age 15 to 89.3% at age 20, identifying late adolescence and early adulthood as a critical period during which women remain at risk of undergoing FGC.

Despite the persistently high prevalence, statistically significant decreases in FGC prevalence were observed among all age groups, with the greatest absolute reduction (15.9% decrease) occurring among respondents aged 15 years. However, comparisons of mean annual rates of change before and after 2013 did not demonstrate statistically significant differences for any age group. Together, these findings suggest that although meaningful progress has been made in reducing FGC prevalence—particularly among younger cohorts—the pace of decline has not accelerated in recent years.

The weighted FGC prevalence of 87.3% observed in this study is consistent with regional estimates. The 2019 Sierra Leone Demographic and Health Survey formal report stated an overall FGC prevalence of 83%; however, that estimate reflects a difference in analytical approach. DHS used the full survey sample (15,574 women) as the denominator rather than restricting the analysis to respondents who provided information on FGC status (14,644 women) [8]. By limiting the denominator to respondents with reported FGC status and applying DHS sampling weights to generate nationally representative estimates, we provide a more accurate estimate of FGC prevalence among women in Sierra Leone.

Moreover, prior DHS-based analyses typically report single cross-sectional prevalence estimates with overlapping age-defined cohorts, which limits accurate comparisons over time [8, 9]. Our temporal analysis incorporated both respondent age at survey and reported age at FGC, enabling reconstruction of annual prevalence estimates from 1984 to 2019 and revealing temporal patterns not apparent in conventional cross-sectional analyses.

These findings have important public health implications given that FGC confers no medical benefit and is associated with both immediate and long-term harm to girls and women [1, 3]. In Sierra Leone, an estimated 85% of girls and women who have undergone FGC have experienced complications, ranging from acute adverse events including excessive bleeding, infection, and pain, to long-term consequences, such as chronic pain, scarring, dyspareunia, and infertility [11, 12]. In recognition of these harms, the United Nations has called for the elimination of FGC as a target with the Sustainable Development Goals [13]. Accordingly, robust estimation of FGC prevalence and temporal trends is essential for monitoring progress towards elimination and for informing evidence-based prevention efforts.

Strengths of this study include the use of a large, nationally representative dataset and the application of a novel temporal analytical approach. By incorporating both respondents’ age at survey and their reported age at FGC, we were able to reconstruct annual estimates of FGC prevalence, from 1984 to 2019, providing a more granular assessment of temporal trends than has been previously reported.

Several limitations related to the analytical assumption of our study should be considered. First, age at FGC was self-reported and may be subject to recall bias, particularly among older respondents; however, any misclassification is likely nondifferential to the overall analysis. Second, differential survival or survey participation related to FGC status could influence estimates. If women who experienced FGC were less likely to survive or participate in the survey, the prevalence for earlier periods could be underestimated. Next, the analysis relied on a single DHS dataset obtained in 2019. As a result, reconstructed age-specific cohorts are retrospective estimates and may not fully represent the population present in earlier decades, especially given population displacement, migration, and death that occurred surrounding the Sierra Leone civil war from 1991 to 2002 [14]. Additionally, the period after 2013 is relatively short, which may limit statistical power to detect changes in temporal trends. The DHS dataset only included girls and women aged 15 to 49, limiting analysis among younger children. Finally, the cross-sectional nature of the underlying data limits causal inference regarding the drivers of observed changes in prevalence; identifying these drivers represents an important area for future research to inform the development of effective prevention strategies.

## Conclusion

The prevalence of FGC in Sierra Leone remains high but has declined over time, with the largest reductions observed among younger age groups. Late adolescence and early adulthood represent a critical period of ongoing risk. Despite significant overall declines in prevalence, no evidence of accelerated reductions was observed following 2013, underscoring the need for targeted interventions at younger ages, when FGC is most commonly performed, as well as during the critical period between ages 15 and 20. Continued investment in prevention strategies, surveillance, and community-based interventions will be essential to achieve meaningful progress toward eradication of this harmful practice.

## Data Availability

The data used in this study are publicly available from the Demographic and Health Surveys (DHS) Program. DHS datasets can be accessed upon request and registration through the DHS Program website: https://dhsprogram.com/data/.

## Declarations

### Previous Presentations

This study was presented as an oral presentation at the American Urogynecologic Society PFD Week in Vancouver, Canada, October 2025.
